# Spider venom phospholipase D toxin structure: Interfacial binding site, mechanism, activation, and head group preference

**DOI:** 10.1073/pnas.2513997123

**Published:** 2026-04-06

**Authors:** Alexandra K. Sundman, Greta J. Binford, William R. Montfort, Matthew H. J. Cordes

**Affiliations:** ^a^Department of Chemistry and Biochemistry, University of Arizona, Tucson, AZ 85701; ^b^Department of Biology, Lewis & Clark College, Portland, OR 97219

**Keywords:** cocrystal structure, phospholipase D, interfacial binding site, interfacial enzyme, loxoscelism

## Abstract

Envenomation by sicariid spiders such as the brown recluse can cause loxoscelism, a syndrome involving localized dermonecrosis and/or systemic effects like hemolysis. The causative venom toxins are unusual interfacial phospholipase D enzymes that cyclize sphingolipid and lysophospholipid substrates when bound to membrane surfaces. Crystal structures of several of these toxins have been reported, but none of them directly illuminates how lipids bind in the active site and at the interfacial binding site (IBS); indeed, as a general rule the lipid interfaces of peripheral membrane proteins resist crystallographic determination. Here, however, we report X-ray crystal structures at 1.85 to 2.6 Å resolution of a venom toxin from the Chilean six-eyed sand spider *Sicarius levii (terrosus)* bound to a micelle-like agglomeration of product and substrate sphingolipids. Each enzyme subunit binds three sphingolipid molecules, one in the active site and two at adjacent noncatalytic sites, generating an interface that approximates the IBS predicted by molecular dynamics. The conformations of substrate and cyclic product in the active site definitively confirm our previously proposed catalytic mechanism. Comparisons with lipid-free structures show conformational changes in two loops that suggest a mechanism for allosteric/surface activation. Docking studies suggest that the variable preference of these toxins for phosphocholine and phosphoethanolamine head groups involves subtle changes in size and shape of the active-site pocket. The structures reveal key facets of the molecular basis of loxoscelism and show that in favorable cases crystallography can illuminate the IBS of peripheral membrane proteins.

## Significance

Recluse spider venoms carry a unique toxin that is not found in other spider venoms and triggers a medical syndrome called loxoscelism, which can involve severe localized skin and tissue damage as well as systemic effects. The toxin is an enzyme that attacks the lipids in cell membranes. Through crystallization and structure determination, we reveal the chemical mechanism and cell–membrane binding surface of these toxins. This knowledge may enable development of small-molecule inhibitors to treat loxoscelism.

Interfacial enzymes are peripheral membrane proteins that catalyze reactions at the boundary between water and a lipid bilayer, and often modify membrane constituents such as phospholipids. These catalysts adhere to membrane surfaces using an interfacial binding site (IBS), a critical feature that not only directs the enzyme to substrate-rich surfaces but may also undergo or trigger activating conformational changes (1). Interfacial enzymes and other peripheral proteins are integral to numerous physiological processes and may act as toxins, making them attractive for targeting by drugs (2), including by interfering with the IBS (3). Unfortunately, the interfaces of peripheral membrane proteins pose tough challenges for experimental structural biology, and are especially intractable to X-ray crystallography. Few high-resolution experimental structures of peripheral membrane proteins bound to lipid surfaces have been reported (4, 5) other than large highly symmetric complexes by cryoelectron methods (6–8). Here, we report an example of a crystallographic structure that illuminates the IBS of an interfacial enzyme.

Sphingomyelinase D/phospholipase D (SMaseD/PLD, or GDPD-like SMaseD/PLD), an interfacial enzyme toxin, is an abundant constituent of the venom of sicariid spiders (9, 10) and a pathogenicity determinant for some species of Actinobacteria (11, 12). SMaseD/PLD is the primary spider venom component responsible for triggering loxoscelism, a necrotic and/or hemolytic syndrome resulting from envenomation of humans by spiders in the *Loxosceles* genus, which includes the brown recluse (10, 13, 14). The enzyme has been experimentally shown to bind the outer plasma membrane of cells (15–18) and cleave choline or ethanolamine head group alcohols from phosphosphingolipid and lysophospholipid backbones (19–23). Primary substrates include sphingomyelin (SM), ceramide phosphoethanolamine (CPE), lysophosphatidylcholine (LPC), and lysophosphatidylethanolamine (LPE), with one study observing activity against additional lysolipids (19, 20). Head group preference varies widely among the spider enzymes, with toxins in the α clade, the group containing the known necrotic and hemolytic variants, generally preferring phosphocholine (PCho) over phosphoethanolamine (PEtn) (19, 20). The bispecificity of the family for PCho/PEtn head groups may relate to the variable presence of both SM (PCho head group) and CPE (PEtn head group) as membrane sphingolipids in invertebrate prey, as opposed to vertebrates where SM dominates (24). Loss of the head group alcohol occurs via nucleophilic displacement at phosphorus by an obligate hydroxyl group in the lipid substrate, leaving behind a cyclic lipid product (25, 26). Membrane remodeling is putatively dependent on enzymatic catalysis (15–18, 27). In particular, the unusual cyclic sphingolipid product cyclic ceramide-1,3-phosphate (CCP) may remodel and damage membrane structure by disrupting lipid rafts (16, 28).

The reaction mechanism, basis of head group preference, and IBS of SMaseD/PLD have yet to be determined experimentally. Crystal structures of three spider toxins revealed an active site with two critical histidine residues and an obligate magnesium ion coordinated by three acidic residues (19, 29–31). None of these structures has lipid substrate/product bound to the enzyme, though one was recently reprocessed to model a cyclic phosphate species in the active site, putatively resulting from an adventitious, catalyzed cyclization reaction involving Tris base and phosphate (32); this finding supports the fundamental cyclization chemistry of the enzyme but does not directly illuminate the mechanism. Early proposed mechanisms invoked covalent catalysis, with an initial attack by one of the histidines at a magnesium-coordinated phosphate (30, 31). Docking studies from our laboratory instead pointed to direct cyclization of the substrate through acid–base catalysis involving the histidines (19), a mechanism similar to that proposed for the distantly related glycerophosphodiester phosphodiesterases (GDPD) (33). Recent QM/MM calculations support this noncovalent mechanism (32). Our docking studies placed the head group deep in the active site pocket, but no clear basis for head group preference emerged, as the residues directly contacting the head group were conserved across the spider toxins. Finally, the IBS was originally proposed to involve two loops (β2α2 or “catalytic” loop, and the β6α6 or “flexible” loop) adjacent to the active site (30), a hypothesis recently supported by molecular dynamics simulations (34).

Here, we report structures of St_βIB1i H47N, a catalytically inactivated version of a toxin from the Chilean six-eyed sand spider *Sicarius levii (terrosus)* (35), bound to substrate (CPE) or product (CCP) at the active site and two noncatalytic sites. St_βIB1i belongs to a spider toxin subgroup called the βI-ABC clade and is specific for substrates with PEtn head groups, such as CPE (19, 36). Remarkably, the protein and lipids crystallize into a lipoprotein complex that appears to recapitulate the postulated IBS. The mode of lipid binding in the active site confirms our proposed catalytic mechanism. Comparison with the structure of the lipid-free wild-type protein (19) shows changes in loop conformation that point to a mechanism of surface/allosteric activation through binding of substrate at noncatalytic sites. Finally, we propose that head group preference is controlled by subtle changes in the active site pocket involving second-shell residues and changes in global structure. This structural study sheds light on the action of recluse spider and bacterial SMaseD/PLD toxins and shows that in fortunate circumstances the interfaces between peripheral membrane proteins and lipid surfaces can be captured in a crystal lattice.

## Results

### Crystal Structures of St_βIB1i-H47N with Bound Lipids.

**Fig. 1.**
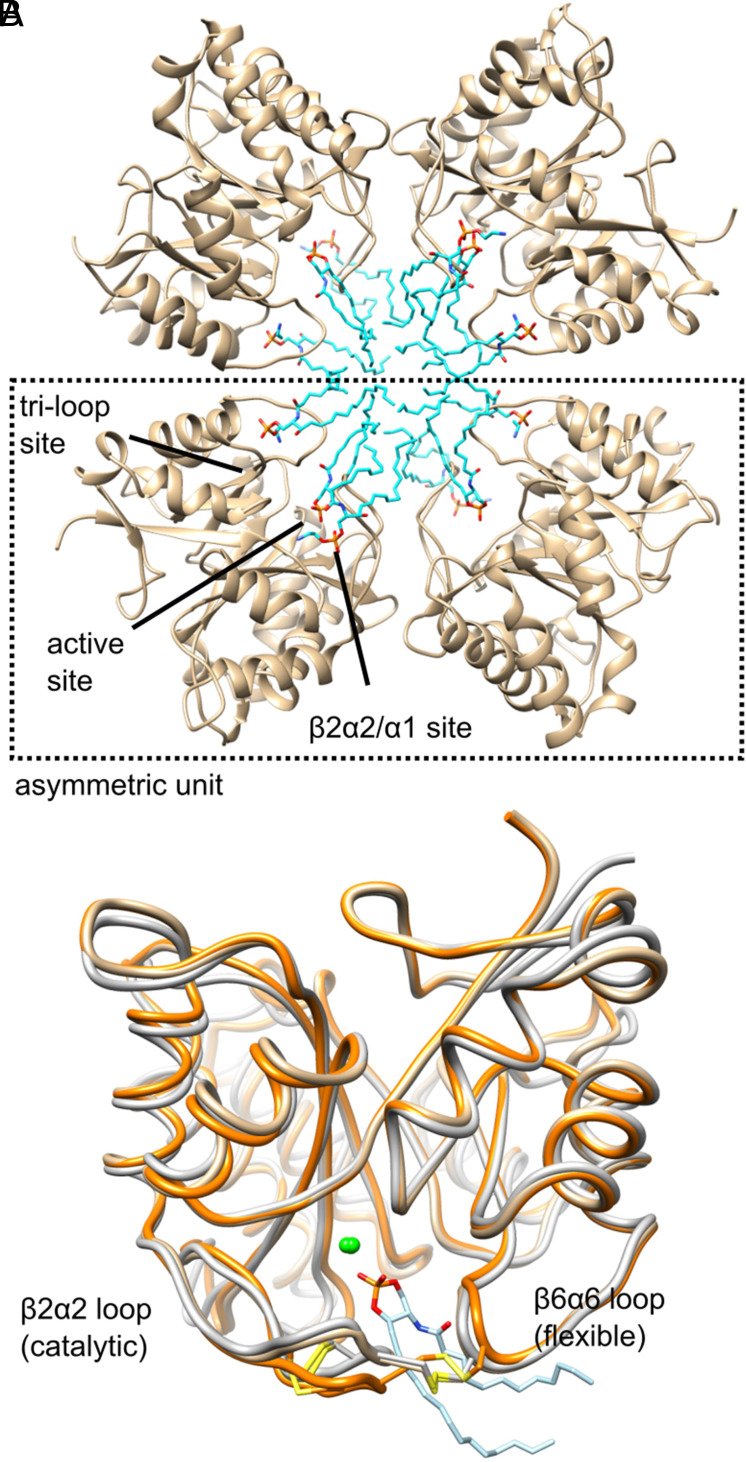
Structure of a St_β1Β1i H47N/lipid complex at 1.85 Å. (*A*) Two copies of the asymmetric unit, which contains two subunits of protein (tan), each of which shows three binding sites for lipids (cyan). The second dimer is generated by symmetry operations −X Y −Z + [0 0 1]. (*B*) Comparison of subunit structure to other class II phospholipase D toxins from sicariid spiders. St_β1Β1i H47N (chain A; tan) is most similar overall to wild-type St_β1Β1i (orange; PDB ID 4Q6X) but more similar to Li_α1Α1 (gray; PDB ID 3RLH) in two loops that contain the two conserved disulfide bonds; CCP product (light blue) is shown bound to the magnesium cofactor (green) in the active site.

We crystallized St_βIB1i in the presence of 0.8 mM synthetic d17:1/12:0 CPE substrate in 10 mg/mL CHAPS detergent, using 16.5% (v/v) 2-methyl-2,4-pentanediol (MPD) as precipitant (*Materials and Methods*). The protein contained a mutation in a catalytic histidine residue (H47N) to enable substrate cocrystallization. Crystals grown for 6 d at ambient temperature diffracted to 1.85 Å resolution in space group *I*_2_, and the structure was determined by molecular replacement using the 2.15 Å structure of wild-type St_βIB1i (19). The model was refined to *R*_crys_ = 12% and *R*_free_ = 17% (*SI Appendix*, Table S1). The asymmetric unit contains two nearly identical protein subunits, each with three lipid molecules bound, one in the active site and two at adjacent noncatalytic sites: one mostly between the β6α6 and β7α7 loops but also engaging the β2α2 loop (“tri-loop site”) and one between the β2α2 loop and helix α1 (Fig. 1*A*). In this structure the active site CPE lipid had been converted to the product cyclic ceramide-1,3-phosphate (CCP) (25, 26) (Fig. 1*B*), owing to weak residual catalytic activity (*SI Appendix*, Fig. S1). All three lipids lacked clear density for the six to seven terminal carbons of the hydrocarbon chains, and the β2α2/α1 CPE molecule was supported only by density for the PEtn head group (*SI Appendix*, Fig. S2).

Isomorphous datasets on crystals grown for 2 d (2.2 Å resolution) and 3 wk (2.6 Å resolution) improved the lipid modeling and contributed a structure with CPE substrate in the active site (*SI Appendix*, Table S1). In the 3-wk dataset, the lipid density in the active site matched better to substrate than to product and was modeled as such (*SI Appendix*, Fig. S2). Loss of catalytic activity over time, coupled with exchange of lipid between sites and from the drop, likely accounts for the repopulation of the active site pocket with substrate in the 3-wk crystal (*Materials and Methods*). Meanwhile the 2-d dataset, like the 6-d dataset, showed product in the active site, but had greatly improved density for lipid in the β2α2/α1 site, allowing it to be clearly identified as CPE (*SI Appendix*, Fig. S2). In other respects, the three structures were extremely similar.

The crystal lattice contains a highly unusual lipoprotein superstructure. Four protein subunits from two asymmetric units surround a discoidal collection of at least 12 partially ordered lipid (CCP or CPE) molecules (Fig. 1*A*). We saw no clear density attributable to CHAPS detergent, suggesting that during crystallization the protein extracted the lipids from the detergent and gathered them into a micelle-like structure. As described in detail below, the lipoprotein complex, though unlikely to represent a biological assembly in a strict sense, likely mimics the core interface between the enzyme and lipids in a native membrane bilayer.

### Comparison to Structures of Homologs.

The structure of St_βIB1i H47N resembles, in different aspects, previously reported structures of two class II (two disulfide bonds) recluse spider phospholipase toxins (19, 29) (Fig. 1*B*). The active site contains the magnesium cofactor conserved in these enzyme toxins. The overall subunit structure resembles that of wild-type St_βIB1i (0.6 Å backbone RMSD), as one might predict, but differs considerably in the β2α2 and β6α6 loops (19). These two loops instead resemble those of another class II toxin, Li_αIΑ1 (aka LiRecDT1) from *Loxosceles intermedia*, even though the overall structure is less similar to Li_αIΑ1 (1.0 Å backbone RMSD) than to wild-type St_βIB1i (29). A similar loop conformation is also found in the class I (one disulfide bond) toxin Ll_A αIII1. As explained further below, the changes in β2α2 and β6α6 loops likely represent conformational activation that is lipid-dependent in St_βIB1i but perhaps not in α clade toxins.

### Catalytic Mechanism.

**Fig. 2.**
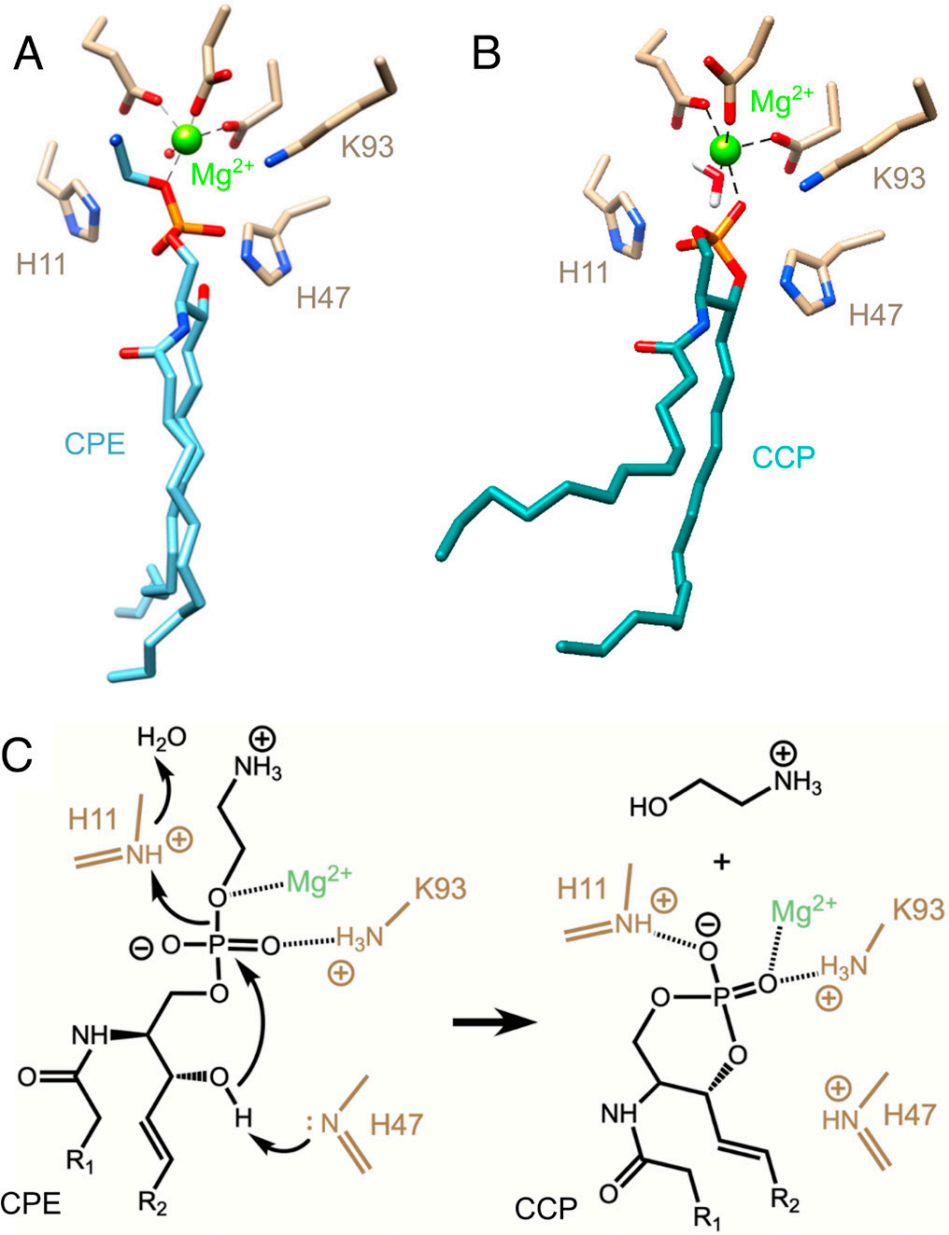
Conformation of substrate and product bound in the active site of St_β1B1i H47N establishes the reaction mechanism. (*A*) CPE substrate (2.6 Å, chain B), with His-47 restored in silico based on the conformation found in Li_α1Α1 (PDB ID 3RLH). (*B*) CCP product (1.85 Å, chain A), with His-47 again restored in silico. (*C*) Proposed catalytic mechanism, wherein His-11 and His-47 act as general acid and base, respectively, while magnesium and Lys-93 coordinate the phosphate moiety.

The binding modes of substrate (CPE) and product (CCP) in the St_βIB1i H47N structure support our previously proposed mechanism (Fig. 2) (19). When the mutated His-47 is modeled back into St_βIB1i H47N, the ε2 nitrogen of the imidazole group is immediately adjacent (2.4 Å) to the nucleophilic hydroxyl group in the CPE substrate (Fig. 2 *A* and *C*) and to the corresponding oxygen atom in the product (Fig. 2 *B* and *C*). This positioning is consistent with the proposed role of His-47 as a general base. The hydroxyl nucleophile is also poised for backside nucleophilic displacement of ethanolamine by attack at phosphorus. His-11, meanwhile, is well positioned to act as a general acid and protonate the leaving group, and the Mg^2+^ cofactor also engages the leaving group oxygen and could stabilize developing negative charge in the transition state. We also note that the H47N mutation in our structures is unlikely to be a major structural perturbation, as docking of substrates to in silico mutated structures with restoration of His-47 give conformations that are very similar and also support this basic mechanism (see below and Fig. 6 below).

This mechanism is closely related to that proposed for the distantly related GDPD enzymes (33), but the orientation of the substrate and the roles of the two histidines are reversed. Hence, this has been called a “reversed orientation” mechanism (10). On a somewhat related note, the cyclic phosphate ring of the CCP product is in an essentially reversed orientation relative to the adventitious cyclic phosphate product recently modeled into the active site of Li_αIΑ1 (32). In fact, a portion of that cyclic phosphate backbone seems to roughly mimic the bound ethanolamine head group of CPE substrate in St_βIB1i (*SI Appendix*, Fig. S3).

### Binding of Substrate Hydrocarbon Chains.

**Fig. 3.**
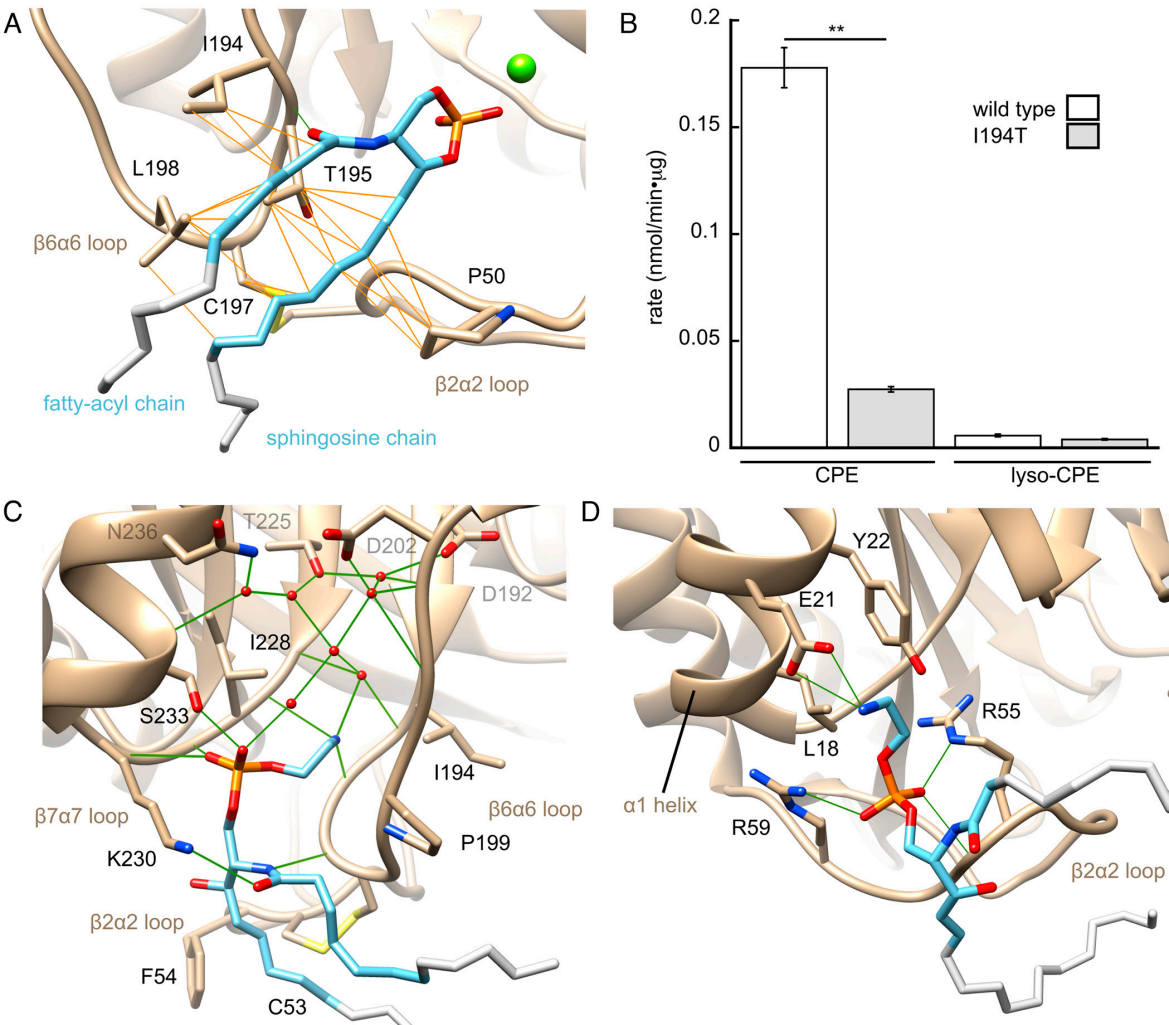
Lipid binding sites of St_βIΒ1i. Catalytic and noncatalytic lipid-binding sites, with well-modeled parts of lipids (light blue) and putatively mobile portions (gray) highlighted. Side chains within 4.5 Å of lipids are labeled and hydrogen bonds shown (green lines). (*A*) CCP bound to active site, with hydrophobic contacts to the acyl and sphingosine chains (carbon–carbon distances <4.5 Å in either/both the CCP- or CPE-bound structures) shown as orange lines. (*B*) Significant effect of I194T mutation on activity against CPE but not lyso-CPE, which lacks the fatty acyl chain. (*C*) CPE bound at the noncatalytic “tri-loop” site; residues involved in water-mediated contacts are also shown (gray labels). (*D*) CPE bound to the noncatalytic β2α2/α1 site. In *B,* data represent mean ± SEM (n = 3). For the effect of the mutation on activity against CPE or lyso-CPE, *P < 0.05 and **P < 0.005, determined by Student’s t test.

The enzyme also makes numerous contacts with the lipid backbone that may strengthen substrate/product binding and ensure proper orientation for catalysis (Fig. 3). The sphingosine chain rests in a shallow groove between the β2α2 and β6α6 loops and makes hydrophobic contacts with β2α2 loop residue Pro-50, along with β6α6 loop residues Thr-195, Cys-197, and Leu-198 (Fig. 3*A*). The fatty acyl chain contacts the hydrophobic side chains of the flexible loop at residues Ile-194, Thr-195, and Leu-198. In addition, the carbonyl oxygen of the fatty acyl chain forms a hydrogen bond (2.9 Å) with the β6α6 loop backbone at residue Thr-195. We suggest that the hydrophobic interactions aid in abstracting lipid from the bilayer and establishing its orientation. The polar contact from Thr-195 may further orient bound substrate in a catalytically competent conformation. These contacts support a major role for the β2α2 and β6α6 loop surfaces in the interfacial lipase activity.

To investigate the contribution of specific hydrophobic β6α6 loop interactions with the fatty acyl chain of substrate, we performed site-directed mutagenesis of β6α6 loop residue Ile-194 and compared the effect on activity against CPE and lyso-CPE, a CPE analogue that lacks the fatty acyl chain. An I194T mutation resulted in a 6.5-fold decrease in rate against CPE, but no significant effect against lyso-CPE (Fig. 3*B*). The effect on CPE (but not lyso-CPE) cyclization is consistent with a primary role for the β6α6 loop in recognition of the fatty-acyl chain of sphingolipid substrates. Whether these interactions contribute primarily through affinity for substrate or by establishing the competent conformation/orientation remains to be seen.

The electron density of bound CCP/CPE lipid chains in the active site is well defined only through 6 to 7 carbons, suggesting that the protein does not immobilize the free ends of the chains through binding and they may remain partly embedded in the bilayer (*SI Appendix*, Fig. S2). Notably, it has been recently reported for Li_αΙΑ1 that the optimal fatty acyl chain length for SM substrate is 6:0, and both shorter and longer hydrocarbon chains are poorer substrates (37). Shorter chains may bind the β6α6 loop poorly, while longer chains may increase affinity of substrate for the bilayer without any balancing interactions to the enzyme.

### Noncatalytic Lipid-Binding Sites and the IBS.

The structure reveals two noncatalytic CPE-binding sites (Fig. 3 *C* and *D*). One CPE molecule is bound with its PEtn head group in a pocket between the β6α6 and β7α7 loops (Fig. 3*C*). The phosphate moiety is held by hydrogen bonds with the side chain of Ser-233 and the backbone of the β7α7 loop. The ethanolamine moiety is positioned deeper in the pocket, with the methylene groups contacting the side chain of Ile-228 and the amine group forming hydrogen bonds to backbone carbonyl oxygens of residues Ser-227 and Thr-195. The PEtn head group also contacts an extensive network of ordered waters deeper in the pocket, which is organized in part by hydrogen bonds to Asp-192, Asp-202, Thr-225, and Asn-236. Meanwhile, the amide functionality of the ceramide backbone is hydrogen-bonded to the backbone of Asn-196 and to the side chain of Lys-230. Finally, the well-modeled parts of the CPE hydrocarbon chains contact Cys-53/Phe-54 and Pro-199 in the β2α2 and β6α6 loops, respectively. The extensive network of interactions between CPE and three loops suggests the name “tri-loop site.” Contacts to the amide and ethanolamine moieties suggest that the tri-loop site of St_βIB1i could be specific for CPE over other lipids.

The CPE molecule in the second noncatalytic site is bound along one side of the β2α2 loop and against helix α1 (Fig. 3*D*). The phosphodiester oxygens make salt bridges to the side chains of Arg-55 and Arg-59 and a hydrogen bond with the backbone amide nitrogen of Ser-56. The ethanolamine moiety makes a salt bridge between its amine group and the side chain of Glu-21 in helix α1, along with contacts to the side chains of Leu-18 and Tyr-22. The lipid backbone, meanwhile, is mostly disordered and shows no clear contacts to the protein. These observations suggest that the β2α2/α1 site is somewhat weaker, and potentially less specific, than the tri-loop site.

**Fig. 4.**
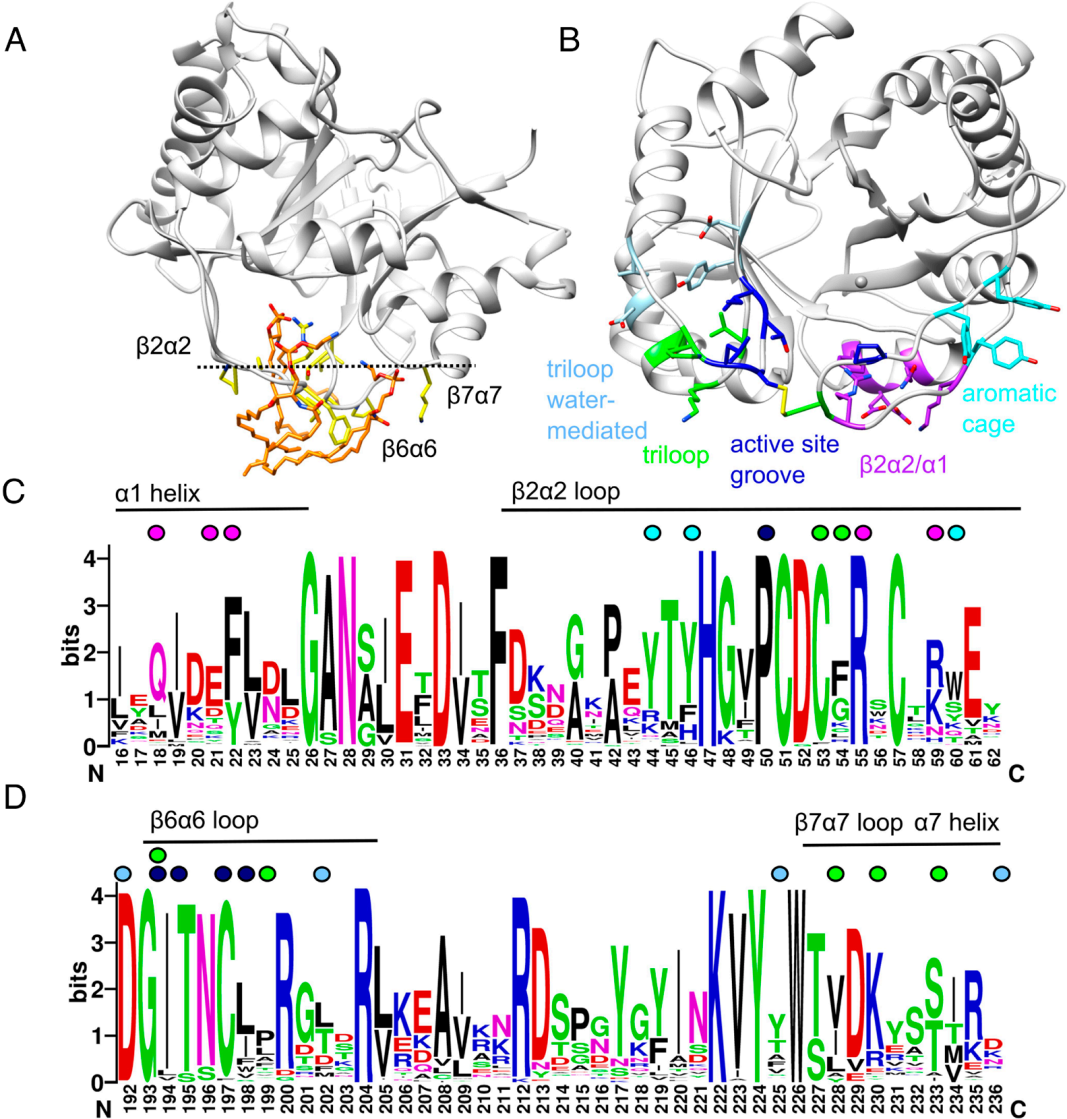
Proposed IBS. (*A*) The lipid–protein interface of each St_βIB1i subunit centers around contacts to the β2α2 and β6α6 loops, both in the crystal structure and in molecular dynamics simulations. Lipids (orange) bound to St_βIB1i in the crystal structure are surrounded by residues (yellow) previously observed to make hydrophobic contacts in a simulation of St_βIΒ1i bound to a POPC/POPE bilayer. The approximate water–membrane boundary in the simulation is indicated by a dashed line. (*B*) Four known lipid binding sites for sicariid toxins mapped onto the structure of Li_α1Α1 (3RLH), including three identified in this study and a fourth (aromatic cage) identified previously using simulations and mutagenesis. (*C* and *D*) Sequence conservation among sicariid toxins for the known lipid-binding sites, with relevant residues indicated by circles color-coded as in *B*.

The three lipid binding sites identified here for St_βIB1i form a contiguous interface that may constitute part or all of the IBS (Fig. 4). The lipid chain contacts to residues in the β2α2 loop (Pro-50, Cys-51, Cys-53, Phe-54, Arg-55), β6α6 loop (Ile-194, Thr-195, Cys-197, Leu-198, Pro-199), and α7 helix (Lys-230) are consistent with hydrophobic protein–membrane interactions previously identified by in silico molecular dynamics of surface binding based on the structure of wild-type St_βIB1i (34). (Fig. 4*A*). The two loops embed shallowly in the membrane (dashed line in Fig. 4*A*) [see also Fig. 5 *C* and *D* of ref. 34]. Predictions of membrane interaction using PPM 3.0 with or without the bound lipids also point to an interface surrounding the β2α2 and β6α6 loops, and suggest that the specific lipid interactions observed in our structures could induce negative curvature in the bilayer (*SI Appendix*, Fig. S4). The lipid binding sites identified here show variable sequence conservation among the sicariid toxins (Fig. 4 *C* and *D*). The active site groove that interacts with the substrate/product lipid chains is very strongly conserved. The tri-loop site tolerates conservative mutations among the residues that contact the PEtn head group, while the deeper part of the pocket is quite variable, as is position 54 which contacts the sphingosine chain. The β2α2/α1 site has well-conserved positive charges that contact the phosphate but is otherwise highly variable in sequence. A fourth site, a PCho-binding “aromatic cage” (Tyr-44, Tyr-46, and Tyr-60 in Li_αIΑ1; see Fig. 4*B*) identified in a previous study using molecular dynamics and mutagenesis, is also quite variable (34). The sicariid toxins, which vary in substrate preference, may also use a shifting repertoire of lipid-binding functionalities to recognize membrane surfaces.

**Fig. 5.**
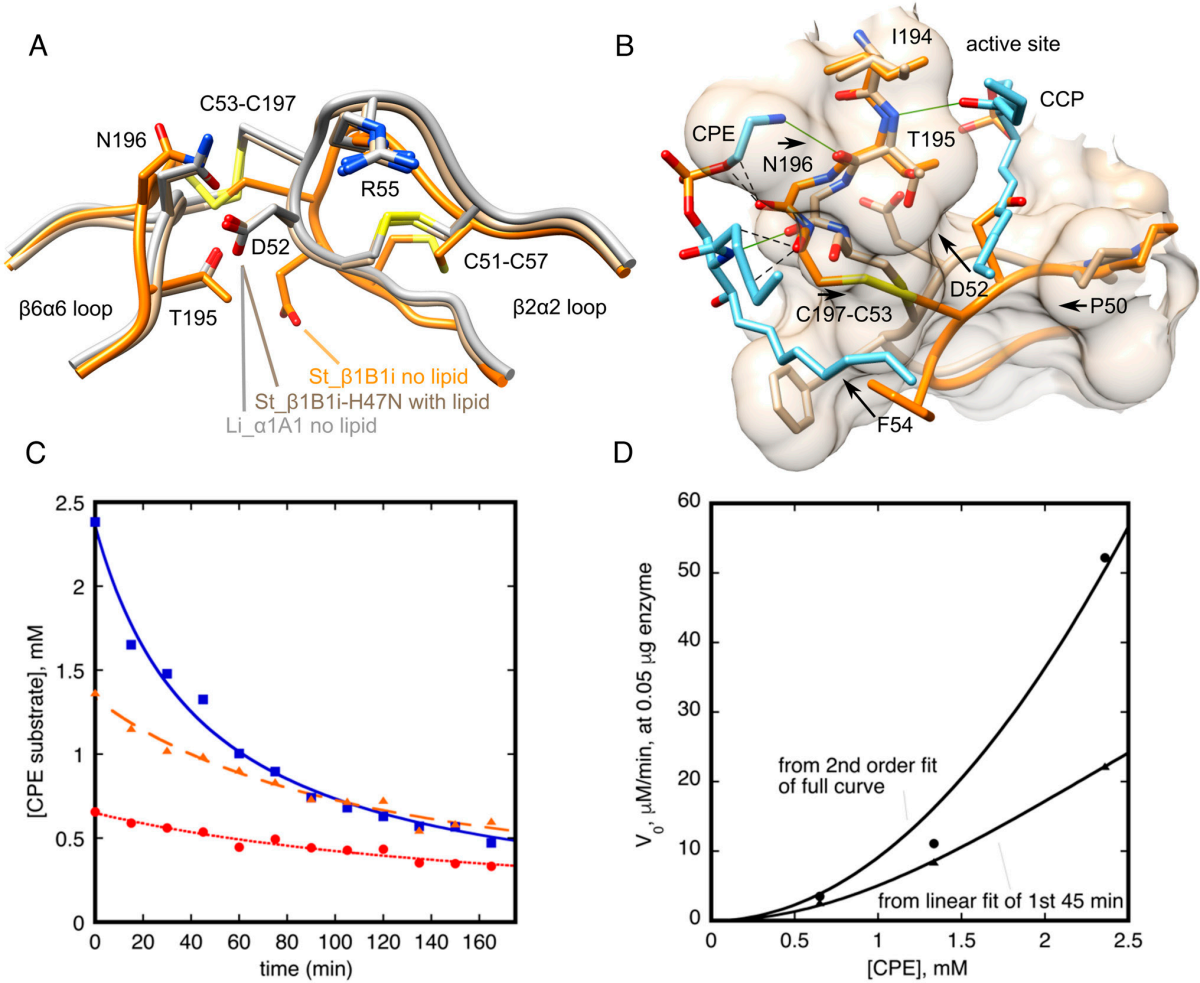
Allosteric activation of St_β1B1i by lipid substrate. (*A*) The β2α2 and β6α6 loops in lipid-free wild-type (orange, PDB ID 4Q6X) and lipid-bound St_β1B1i-H47N (tan) have different conformations. The loops in lipid-bound St_β1B1i-H47N resemble those in lipid-free wild-type Li_α1Α1 (gray, PDB ID 3RLH), suggesting that the loop shift in St_β1B1i is not caused by the H47N substitution. (*B*) Model for activation of St_β1B1i in which CPE bound at the tri-loop site triggers the loop shift and enables lipid binding in the active site. CPE is predicted to clash with carbonyl oxygens of N196 and C197 in the β6α6 loop of wild-type St_β1B1i (dashed lines show interatomic distances ≤ 2.5 Å). In the CPE-bound St_β1B1i-H47N structure the loop is shifted rightward, relieving these clashes and establishing a hydrogen bond (green line) between the carbonyl of N196 and the amide of CPE. Movement of β6α6 leads to a shift in β2α2, communicated in part through the C197-C53 disulfide linkage. Side chain movements in β2α2 open a groove for binding of a sphingosine hydrocarbon chain in the active site. Movement of F54 may also be associated directly with binding of the allosteric lipid near the double bond of the sphingosine chain. The last six carbon atoms of the lipid chains are not shown for simplicity. (*C*) Substrate loss at three different initial substrate concentrations as monitored by ^31^P NMR (*Materials and Methods*). (*D*) Initial velocities, extracted either from fitting of the full kinetic traces to a 2nd order decay (curve fits in *C*), or from the slope of a linear fit of the first 45 min of the run. These data were fit to a cooperative version of a Michaelis–Menten equation with a cooperativity coefficient fixed at 2. In these fits the highest concentration used (~2.5 mM) is well below *K*_0.5_ such that the velocity varies approximately as the square of substrate concentration. The apparent second-order decay observed in the full traces of *A* likely results from this dependence (though in general such a decay profile could have contributions from other sources such as product inhibition). The accessible range of initial substrate concentrations in these experiments is narrow due to issues of solubility and sensitivity, but the initial velocities clearly deviate from standard Michaelis–Menten kinetics and are consistent with allosteric activation by cooperative binding of substrate to the active site and a noncatalytic site.

### Allosteric/Surface Activation.

St_βIB1i H47N with lipids bound has β2α2 and β6α6 loops that adopt markedly different structures from the wild-type and similar to those found in lipid-free α-clade enzyme structures such as Li_αIΑ1 (Fig. 5*A*). Structural superpositions also indicate that both CCP/CPE bound in the active site and CPE bound in the tri-loop site are sterically incompatible with the wild-type loop conformations (Fig. 5*B*). These observations strongly suggest that the wild-type structure represents a low activity conformation, while the lipid-bound St_βIB1i H47N and Li_αIΑ1 structures represent an allosterically activated or surface-activated form. We propose that substrate binding at the tri-loop site promotes loop conformational changes and opens a shallow groove near the active site, in which the sphingosine chain of substrate binds (Fig. 5*B*). In contrast to this βI-ABC clade enzyme, some α-clade enzymes may be constitutively active, adopting the activated form without lipid bound (Fig. 5*A*).

Kinetic measurements are also consistent with CPE-mediated activation in St_βIB1i (Fig. 5 *C* and *D*). In a previous study, we found that an St_βIB1i E134P/G191S variant had some activity against SM substrate in ^31^P-NMR assays, but only in a competition assay with CPE, and only while some CPE substrate remained (19). Here, we find moreover that wild-type St_βIB1i does not obey Michaelis–Menten kinetics in mixed CPE/CHAPS micelles. Initial rates against CPE have a roughly second-order dependence on CPE concentration in ^31^P-NMR assays (Fig. 5*D*); individual kinetic traces show second-order decay that could result from the same concentration dependence (Fig. 5*C*). Such behavior is consistent with substrate cooperativity in a regime where [S]_init_ < *K*_0.5_. Notably, most kinetic studies on α-clade toxins report obedience to Michaelis–Menten kinetics, supporting the idea (see above) that they may be constitutively active (20, 37, 38). In summary, we suggest that CPE substrate bound in the St_βIB1i tri-loop site acts as an allosteric activator or, viewed slightly differently, as a component of a surface activation process mediated by a substrate-containing bilayer.

### Lipid Head Group Specificity.

**Fig. 6.**
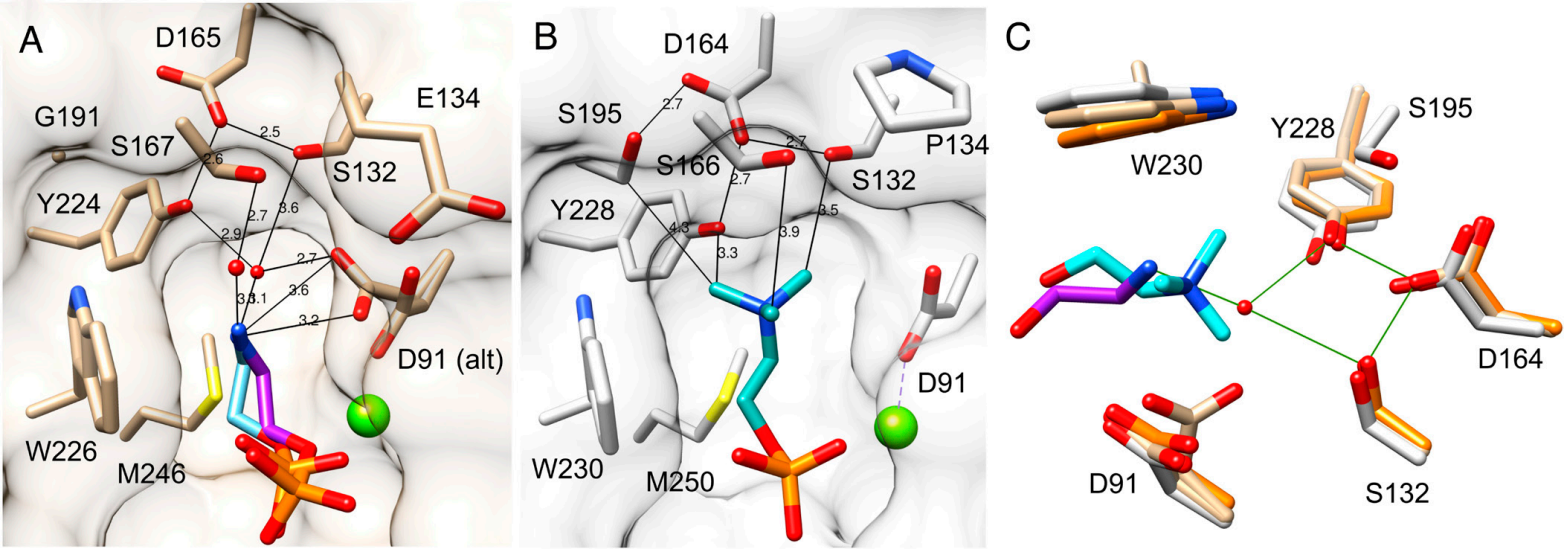
PCho vs. PEtn head group binding. (*A*) CPE bound to St_β1B1i H47N (2.6 Å; chain B) with only the PEtn head group shown (purple), compared to the results of in silico docking (light blue). The alternate conformations of D91 may partly reflect ion-pair formation with the primary ammonium group of PEtn. (*B*) Docking of SM to Li_α1A1 (PDB ID 3RLH), with only the PCho head group shown (cyan). (*C*) Overlay of selected head group pocket residues from Li_α1A1 with docked SM (gray and cyan), free wild-type St_β1B1i (orange; PDB ID 4Q6X), and St_β1B1i H47N with CPE bound (tan and purple), with hydrogen bonds involving one bridging water shown (green). Residue numbers are from Li_α1A1. In both free and substrate-bound St_β1B1i, the pocket is narrower at its mouth (D91 across to W230 in *A* and *B*) relative to Li_α1A1, but also slightly deeper (position of the S132/D164/Y228 interacting triad in *C*), differences which may favor the narrower PEtn head group while allowing a bridging water to fit deep in the active site. The peripheral residue S195 in Li_α1A1 may play a role in the precise positioning of the triad, and may directly contact PCho as shown in *B*.

An abiding mystery of the recluse spider phospholipase toxins is why St_β1B1i, a member of the β1-ABC clade of toxins, strongly favors the PEtn head group found in CPE, while α-clade enzymes such as La_α1B2bi favor the PCho head group found in SM (19). Members of both clades conserve the same core constellation of side chains in the head group binding pocket (Fig. 6), with systematic variation observed at two positions on the periphery (19, 36). A double mutation at these positions in St_β1B1i (E134P/G191S) led to clearly detectable SMase activity (19), and we report here that it also decreases CPEase activity by an order of magnitude (*SI Appendix*, Fig. S5). Despite these effects, St_β1B1i E134P/G191S still prefers CPE to SM (19), implying that the determinants of head group preference are not fully explained by the identities of residues in the pocket.

We docked cognate sphingolipid substrates to Li_αIΑ1 and St_β1B1i as representatives of the α and βI-ABC clades, respectively. A low energy pose of CPE docked to St_βIB1i H47N, with His-47 reintroduced by in silico modeling, closely reproduced the head group binding mode seen in our crystal structure of H47N bound to substrate (Fig. 6*A* and see also *SI Appendix*, Fig. S6). The lowest energy pose of SM docked to Li_αIΑ1 was also closely comparable (Fig. 6*B* and see also *SI Appendix*, Fig. S6). The conserved residues in the head group pocket appear to have an interaction repertoire that supports both PCho and PEtn binding. First, Trp-226 is positioned to make a π-cation interaction with either ammonium group. Second, three conserved hydroxyl groups (Ser-132, Ser-167, and Tyr-224, in St_βIB1i numbering) make weak C-H…O hydrogen bonds to the three methyl groups of PCho (39), but also interact with the primary ammonium group of PEtn through water-mediated interactions. Third, the side chain carboxylate of Asp-165, if deprotonated as expected, is positioned to engage the hydroxyl protons of Ser-132 and Tyr-224, so that their lone pairs point toward the pocket for optimal orientation toward the electropositive ammonium groups of both substrates. Finally, Met-246 makes hydrophobic contacts to the methylene groups common to PCho and PEtn.

Despite this intrinsic bispecificity, subtle changes in pocket shape may favor one substrate over the other. The active-site pocket of St_βIB1i is notably narrower at its mouth than that of Li_αIΑ1 and may sterically discourage PCho binding, with the caveat that we were readily able to dock SM into the pocket in a pose similar to that found for docking to Li_αIΑ1, dependent on which of two alternate conformations of Asp-91 was used (compare Fig. 6 *A* and *B*). In addition, subtle shifts in residues deeper in the pocket, such as Tyr-224 and Asp-165, may help accommodate the extra space required by the water-mediated contacts to PEtn (Fig. 6*C*). A switch from glycine to serine at 191 at the periphery may affect the precise positioning of Asp-165 and enable a weak direct contact to the PCho methyl groups. But the preference for PCho versus PEtn may partly depend on subtle shifts in global protein structure that reshape the pocket.

### Sodium Ions.

Finally, we note that in addition to the highly conserved magnesium ion cofactor, the active site of St_βIB1i H47N unexpectedly contains one to two coordinated sodium ions (*SI Appendix*, Fig. S7). No sodium ions were modeled for the wild-type St_βIB1i structure or any other published recluse spider toxin structure, but these other structures were obtained from crystals grown with little to no sodium in the drop (19, 29, 31). One sodium ion occupies a space deep in the head group-binding pocket and is coordinated by Ser-132 and Ser-167, but it does not appear to be incompatible with head group binding of CPE, nor does variation in sodium ion concentration affect St_βIB1i activity (*SI Appendix*, Fig. S7).

## Discussion

The structures described here provide a structural basis for the catalytic mechanism, cyclic product, IBS, conformational activation, and head group preference of interfacial phospholipase D toxins in sicariid spiders. The work is a major breakthrough in the understanding of the function of this medically important toxin family. The presence of an extensive protein–lipid interface in a crystal structure of an interfacial enzyme is striking and surprising. For most peripheral membrane proteins, including classic interfacial enzymes like phospholipase A_2_, the IBS resists high-resolution experimental methods and is instead interrogated with a diverse toolkit (40) including spin-labeled NMR (41), mass spectrometry (42), prediction, and simulation (43, 44). Recent progress with cryoelectron methods mostly involves tomography applied to large, symmetric complexes (6–8); two cryo-EM structures of moderate resolution have been reported for smaller, nonsymmetric peripheral proteins including the prothrombinase complex (5.3 Å) and AP2 clathrin adaptor complex (3.3 Å) bound to nanodiscs (4, 5).

The IBS described herein involves the active site and two noncatalytic sites and resembles the core IBS identified in molecular dynamics simulations for St_βIB1i bound to a POPC/POPE bilayer (34). It is important to note that the IBS identified for Li_αIΑ1 in that study overlapped only partially with that of St_βIB1i, and the orientation of the enzymes on the membrane is variable. For example, Li_αIΑ1 contains an “aromatic cage” motif that aligns to residues R44/K60/S62 in the β2α2 loop of St_βIB1i, near but separate from the β2α2/α1 site described here (Fig. 4*B*). The cage is largely conserved in members of the α clade, and was found to interact with PCho head groups in simulations. Introduction of the cage into St_βIB1i by an R44Y/K60Y/S62Y mutation allowed it to bind SM-containing membranes in simulations in an orientation more similar to that of Li_αIΑ1, and also increased its experimental affinity toward SM-containing liposomes (34). Meanwhile, the tri-loop pocket of St_βIB1i identified here has interactions that are specific to PEtn head groups (and contacts to the sphingolipid backbone), but also shows substantial sequence variation across the family. This family of toxins may use a shifting repertoire of noncatalytic sites to form an IBS, and these sites could have head group (and lipid backbone) preferences that potentially contribute to the overall catalytic specificity, including through selectivity filtering and differential allosteric activation. Going forward, the coevolution of binding preferences of the active site and other sites in the IBS merits deeper exploration, as does comparison to other membrane-binding protein families that distinguish between SM (PCho) and CPE (PEtn), such as the mushroom aegerolysins (45).

The noncatalytic sites may anchor the enzyme to the membrane and promote processive catalysis. The sicariid toxins appear to adhere to membranes (15–18), and at least one previous study found evidence for a processive (scooting) mode of catalysis on membrane surfaces for a toxin from *Loxosceles laeta* (28).

Allosteric/interfacial activation of a lipid-anchored enzyme is a recurrent theme in interfacial phospholipases such as PLA_2_, PI-PLC, and PLD (1, 46–49). St_βIΒ1i shows substrate cooperativity, and CPE substrate also increases the activity of the protein toward noncognate substrates such as SM (19). Allosteric activation through specific binding of CPE to the tri-loop site, leading to opening of a lipid-binding groove at the active site entrance, nicely explains these observations. Interesting comparisons here are PI-PLC, where binding of phosphatidylcholine molecules to specific sites on the enzyme anchor it and promote its allosteric/interfacial activation through motions of surface loops that open the active site (50); and PLA_2,_ which also features an active-site opening mechanism (51).

A caveat to our own results is that conformational activation is not strictly necessary for the observation of substrate cooperativity, and other interactions could also contribute. First, although the electron density is poor for the ends of the lipid chains, it is very likely that there are contacts between the lipid chains of CPE molecules bound at the active site and the noncatalytic tri-loop site. Such contacts could increase substrate affinity for the active site. Second, formation of a multisubstrate complex may accelerate catalysis through rapid exchange between noncatalytic and catalytic sites. We also note that conformational activation may not be a conserved feature of the sicariid toxins, since cooperative kinetics have not previously been reported, and lipid-free Li_αIΑ1 adopts the putative activated conformation we observe in St_βIΒ1i (29).

The catalytic mechanism (Fig. 2) was proposed by us previously on the basis of the wild-type structure (19) and is related to the putative mechanism of the first reaction step for the anciently related GDPD family (33). The common core features of these mechanisms are coordination of the phosphate by a divalent metal ion cofactor, and use of a pair of histidines as general acid and base groups to protonate the leaving group and deprotonate the nucleophile, respectively. One major difference is that the general acid and base roles played by the two catalytic histidines are switched, reversing the orientation of the phosphodiester substrate in the active site. In addition, for GDPD enzymes the magnesium cofactor is proposed to interact with the nucleophilic oxygen and one other phosphate oxygen, whereas for GDPD-like SMaseD/PLD enzymes, magnesium primarily engages the leaving group oxygen and (weakly) one other phosphate oxygen. This latter mode of substrate binding (and a similar overall mechanism) is found in some one-metal ion dependent nucleases (52), for example the T4 endonuclease VII N62D mutant complexed to a DNA Holliday junction (53).

The reversed substrate orientation might be associated with an evolutionary shift from nonlipid glycerophosphodiester substrates (GDPD family) to lipid substrates (GDPD-like SMaseD/PLD family) (54). In the reversed orientation, the lipid backbone and tails face outward from the active site pocket, where they may remain partially associated with a membrane bilayer. The flip may also help explain why GDPD enzymes also catalyze a second, hydrolytic step, whereas a cyclic phosphodiester is the final product for GDPD-like SMaseD/PLD enzymes (25). In GDPD enzymes, the glycerol moiety containing the nucleophile is proposed to be deep in the active site pocket, while for GDPD-like SMaseD/PLD enzymes the alcohol leaving group occupies this position. Consequently, the cyclic intermediate for GDPD substrates is bound more deeply and is susceptible to attack by an incoming water nucleophile.

Our elucidation of substrate orientation and conformation may also aid structure-based development of orthosteric or allosteric inhibitors. The substrate-like molecule edelfosine, an analog of LPC in which the nucleophilic hydroxyl group in the substrate is methylated, is a weak competitive inhibitor of a PLD toxin in vitro (20). In silico screening of small molecules has identified six candidate PLD inhibitors, including the antiparasitic drug suramin, that exhibit low to mid-µM binding affinity (38, 55). However, much work is required to improve the potency and bioavailability of SMaseD/PLD inhibitors.

## Materials and Methods

### Materials.

All phospholipid substrates were purchased from Avanti Polar Lipids (Alabaster, AL). Amplex enzyme assays used natural CPE from porcine brain, synthetic 14:0 LPE (1-myristoyl-2-hydroxy-*sn*-glycero-3-PEtn), and synthetic d18:1 lyso-CPE (D-erythro-sphingosyl PEtn). NMR and crystallographic studies used synthetic d17:1/12:0 CPE (N-lauroyl-D-erythro-sphingosyl PEtn). QIAprep Spin Miniprep kits and nickel nitrilotriacetic acid resin were purchased from Qiagen (Hilden, Germany). QuikChange site-directed mutagenesis kit was purchased from Agilent (La Jolla, CA). BugBuster Protein Extraction Reagent, Benzonase nuclease, and Amicon Ultra centrifugal filtration units (10K MW cutoff) were purchased from MilliporeSigma (Burlington, MA). Amplex Red Sphingomyelinase Assay Kits were purchased from Invitrogen (Eugene, OR), and tyramine oxidase (from Arthobacter *sp.*) was purchased from Sekisui Diagnostics (Burlington, MA). All other reagents were purchased from standard sources.

### Cloning and Mutagenesis.

Expression plasmids encoding the mature toxin sequence of St_βIB1i from *S. *levii* (*terrosus*)*, and E134P/G191S mutations therein, were constructed previously (19). These plasmids were based on pHis8, a modified pET-28a expression vector that supplies an N-terminal octahistidine affinity tag (56). H47N and I194T mutations were introduced into the wild-type plasmid using a QuikChange mutagenesis kit (Agilent, La Jolla, CA) according to the manufacturer’s protocol, and verified by capillary electrophoresis sequencing at the University of Arizona Genetics core facility. Residue numbering used in text and figures in this study reflects actual numbering from the putative first residue of the mature toxin sequence, whereas in prior studies (19) we used a more universal numbering system based on alignment to the sequence of SMaseI from *L. laeta* (31).

### Protein Expression and Purification.

N-terminally octahistidine tagged recombinant proteins were expressed in *E. coli* strain BL21(λDE3), purified using immobilized metal affinity chromatography, and dialyzed into 1× TBS buffer (0.1 M Tris [pH 8.0], 0.2 M NaCl) closely following published methods (19, 26, 57). Protein for crystal screening was further purified by size exclusion chromatography on a Sephacryl S-100 16/60 column equilibrated in 1× TBS. Size-exclusion purified protein was concentrated and exchanged into a buffer with reduced ionic strength (0.25× TBS) using Amicon 4 or Amicon 15 10K MW cutoff centrifugal filters. Protein concentration was estimated from absorbance measurements at 280 nm using the estimated absorbance of tryptophan and tyrosine residues (58). Purified protein was stored in small aliquots at −80 °C following flash-freezing in liquid nitrogen. Aliquots were rapidly thawed in tepid water shortly before use.

### Crystal Structure Determination.

Rod-shaped crystals of St_βIB1i (H47N) complexed with d17:1/12:0 CPE substrate were grown by the hanging drop method at 23 °C. Substrate (d17:1/12:0 CPE) was solubilized to a concentration of 4 mM in a solution of 1× Reaction Buffer (0.1 M Tris [pH 7.41], 10 mM MgCl_2_) containing 50 mg/mL CHAPS detergent. Directly before use, substrate stock was equilibrated at 45 °C for 15 min with vortexing every 2 to 3 min, then centrifuged 10 min at 18,000 g to remove impurities. Substrate solution was combined with purified St_βIB1i stock (2.5 mg/mL protein in 0.25× TBS) in a ratio of 2:8 for working concentrations of 2 mg/mL protein with 0.8 mM CPE. This solution was combined 1:1 with precipitant solution [16.5% methyl-2,4-pentanediol, 60.5 mM NaCl, and 30.25 mM NaOAc (pH 4.6)] for a total drop volume of 3 µL. For data measurement, a crystal was transferred to a precipitating solution supplemented with 21% 2-methyl-2,4-pentanediol as a cryoprotectant, mounted in a measurement loop, and flash frozen in liquid nitrogen. Diffraction data were measured at 100 K using a Bruker Venture D8 MetalJet X-ray diffractometer with Photon III detector. Intensities were integrated and scaled with the Bruker Proteum3 software suite, and structure factor amplitudes estimated with the CCP4 program suite (59). Crystals were in space group *I*2 with unit cell parameters *a* = 80.0 Å, *b* = 105.3 Å, *c* = 108.4 Å, β = 3.8°. A structure solution was obtained by molecular replacement using MOLREP (60) and a starting model prepared from PDB entry 4Q6X (19). Model parameters for St_βIB1i were prepared using the Lydia Ligand Builder in COOT (61) to generate a SMILES string and optimized using ACEDRG (62) in CCP4. Model building was done with COOT and model refinement with REFMAC (63) as implemented in CCP4. Figures were prepared with Chimera or ChimeraX (64).

### Modeling of Ions and Other Small Solutes.

Magnesium ions were modeled in the active site based on the known dependence of the enzyme on this metal, and on the observation of fairly regular octahedral coordination with an average ligand distance of 2.0 to 2.1 Å. Two other putative metal binding sites per subunit were modeled as sodium ions based on coordination by at least five oxygen atoms at a distance of ≤2.7 Å, along with the lack of calcium, the major alternative ion, in the drop (*SI Appendix*, Fig. S7). Modeling as sodium yielded reasonable *B*-factors and no residual difference peaks (Fo-Fc) in the final maps. Finally, one molecule of the precipitant 2-methyl-2,4-pentanediol was modeled per subunit, sandwiched between two subunits and near the α2β3 loop in one and the terminus of the α7 helix in the other.

### Modeling of Lipids.

Active site lipid in the 1.85 Å structure was unambiguously assigned as the product cyclic-1,3-ceramide phosphate (CCP). The product was modeled at full occupancy in both subunits, though substantial negative difference density was observed near the phosphorus atom, and a collection of positive peaks in one subunit (chain B) suggested a minor population of a network of three water molecules in place of CCP, including one coordinated to the magnesium ion. Lipid density in the tri-loop site was clearly assignable to CPE substrate, while that in the β2α2/α1 site was more ambiguous, with prominent phosphate (or sulfate, e.g. from CHAPS detergent) density visible but very weak density elsewhere. Assignment of this lipid to CPE was resolved using the 2.2 Å dataset, which clearly showed the entire PEtn headgroup as well as a characteristic branching at carbon-2 of the sphingosine backbone. For all lipid molecules, very weak density for the ~6 carbon atoms at the chain termini made tracing of the hydrocarbon chain ends challenging. For chain A, the sphingosine chain of CCP was the most readily traced hydrocarbon chain, while for chain B, it was the acyl chain of CCP. Given that the lipid chains occupy a common space in the crystal, indicative of a micelle-like complex, the weak density likely results from chain mobility in the hydrocarbon center of the complex.

### Refinement of Lower-Resolution Structures.

Structures derived from lower-resolution datasets (2.2 Å, 2 d crystal growth; 2.6 Å, 3 wk crystal growth) were refined beginning from the high-resolution coordinates, with removal or adjustment of alternative conformations and solvent as deemed appropriate. Sodium ions at a site deep in the active site pocket were retained from the 1.85 Å structure, though they displayed somewhat weaker coordination geometry; sodium ions at a second site coordinated by the β2α2 loop were removed from the lower-resolution structure based either on a complete absence of density or a movement of the density peak to a location less consistent with sodium.

For the 2.2 Å structure, the most obvious difference from the 1.85 Å structure was improved density for CPE in the β2α2/α1 site. Despite the improved density, a major ambiguity that remained was the placement of the acyl chain and sphingosine backbone, as the density beyond the carbon-2 branch point did not readily distinguish them. The 2.2 Å structure also showed a simpler active site configuration, particularly with regard to the lack of an alternate conformation for Asp-91, with a carboxylate that is tilted and slightly displaced from the magnesium ion relative to the observed conformation in the wild type.

For the 2.6 Å structure, the major difference was that the density for the active site lipid was a poorer match for CCP, particularly in a relative lack of density for the oxygen coordinated to magnesium, and in a continuous stretch of density extending into the head group-binding pocket. Lipid-associated water molecules also showed movements relative to the 1.85 Å and 2.2 Å structures. These changes were most evident in chain B. We considered two possibilities: 1) the cyclic product was being replaced by exchange with substrate (CPE) into the active site as the enzyme lost activity over several weeks; 2) the cyclic ceramide phosphate product was being slowly converted to ceramide-1-phosphate (C1P) by hydrolysis. In terms of modeling the density, we compared CPE versus C1P plus two water molecules in place of the CPE headgroup, and both fit reasonably well. Substrate exchange is plausible for the following reasons. First, the lipid in the triloop site clearly remains as CPE after 3 wk of incubation, suggesting that the drop is not depleted of a reserve of substrate, although exchange involving the triloop site could also be very slow. Second, in ^31^P NMR assays with other toxin variants in CHAPS micelles, we have found that the cyclic product is ≥95% stable toward hydrolysis for at least a month at 37 °C, even at enzyme concentrations where substrate is consumed essentially instantaneously. We also typically observe some loss of activity in enzyme samples stored at 4 °C over weeks to months. Finally, we note a particularly prominent tilted conformation of Asp-91 in chain B of the 2.6 Å dataset, a movement consistent with formation of a salt bridge with the amine group of substrate in the active site (Fig. 6*A*). On the other hand, slow hydrolysis of cyclic product in the active site is also plausible. In the 1.85 Å and 2.2 Å structures, a magnesium-bound water molecule is well positioned 3 Å away for in-line nucleophilic attack on the phosphate cyclic product. If the product exchanges very slowly in the crystal lattice, it will remain constantly vulnerable to hydrolytic attack, a situation which may not exist in solution. It is also possible that the H47N variant has unusual properties with respect to cyclic product hydrolysis. On balance, we elected to model the density as substrate, with the caveat that hydrolytic product cannot be rigorously excluded.

### In Silico Docking.

Docking of CPE to St_βIB1i H47N and SM to Li_α1A1 (PDB ID 3RLH) was performed with Autodock Vina (65, 66). Proteins were prepared for docking using MGLTools (https://ccsb.scripps.edu/mgltools/). For St_βIB1i H47N, the high-resolution (1.85 Å) product-bound structure was used as receptor to avoid biasing the substrate binding, and Asn-47 was mutated in silico to histidine with a side-chain conformation close to those observed in Li_α1A1 and wild-type St_βIB1i. In general, water molecules and ions were removed with the exception of the catalytic magnesium ion. Protonation states of catalytic histidines were set based on the assumption that His-47 uses its ε2 nitrogen as a general base and His-11 acts as a general acid. To reduce search space, the linear hydrocarbon chains of SM and CPE substrates (d17:1/12:0) were restricted to the dihedral angles observed in the St_βIB1i H47N structure while all other dihedral angles were permitted to vary. In the docking search, the lipid substrate was confined to a 10 Å by 17 Å by 22 Å box centered to encompass the region occupied by the lipid chains in the St_βIB1i H47N structure, plus the head group binding pocket. 50 poses each were generated for Li_α1A1/SM and St_βIB1i/CPE with an exhaustiveness setting of 32 (highest), and analyzed using ChimeraX. For St_βIB1i/CPE, the docking results were largely insensitive to 1) the precise conformation of the modeled His-47 side chain (varied between −170 and −180 for χ1 and −60 to −70 for χ2), 2) the choice A or B subunit, 3) the choice of alternate conformations for Asp-91 and Met-246 in the active site, 4) the presence or absence of an active site sodium ion. In all runs, a CPE pose comparable to that observed for Li_α1A1/SM was found with an energy between 0.0 to 0.5 kcal/mol of the lowest energy pose. The only competing family of docking poses, which we rejected, was similar but with the phosphate group moved away from the magnesium ion (*SI Appendix*, Fig. S6).

### Enzyme Kinetics.

Head group release assays with CPE and lyso-CPE in Triton X-100 micelles were performed with an Amplex Red Sphingomyelinase Assay Kit essentially as described previously (57), except that for substrates with PEtn head groups, the secondary enzyme choline oxidase (0.1 U/mL) is substituted with tyramine oxidase (2.5 U/mL) to detect ethanolamine rather than choline release (67–69). Partially limiting rates of secondary enzymatic reactions were accounted for in data fitting by modeling the combined secondary reactions as a first-order process, the rate constant for which was measured independently by directly supplying ethanolamine as a substrate for the secondary enzymes. Unless otherwise noted, nominal initial substrate concentrations were 200 µM, and reactions were performed in 0.1 M Tris (pH 7.4), 10 mM MgCl_2_, 0.1% Triton X-100 at 37 °C. Rates for St_βIB1i H47N were measured with 20 µg of enzyme in paired reactions with a negative control lacking enzyme. St_βIB1i wild type and I194T samples were compared in paired reactions. To elucidate the role of sodium concentration, wild-type St_βIB1i samples were run against CPE in paired reactions with and without 0.2 M NaCl. ^31^P-NMR spectra were recorded at 310 K on a Bruker DRX-500 spectrometer equipped with a BBO-500 MHz S2 5 mm probe with a Z gradient. Samples containing 0.6 to 2.4 mM of synthetic d17:1/12:0 CPE were prepared in 1× Reaction Buffer (100 mM Tris [pH 7.4], 10 mM MgCl_2_) containing 50 mg/mL CHAPS detergent, 10% deuterium oxide, and 1 mM trimethyl phosphate (TMP), and treated with 50 ng enzyme. ^31^P-NMR spectra were obtained at 15 min intervals over several hours at 310 K using 104 transients, a 7 μs pulse width, and a 7 s relaxation delay. Peak areas for CPE and CCP were obtained from line shape simulations using MestReNova software and converted to concentrations based on comparison with the peak area for the 1 mM TMP standard.

### Sequence Conservation Logos.

Residue conservation was assessed using a dataset of 99 GDPD-like SMaseD/PLDs that maximize phylogenetic and taxonomic breadth within the venom-expressed lineage. Sequences were aligned using MAFFT (70), curated to exclude >90% identity with MMseqs2 (71) and visualized using WebLogo V2.8.3 (72) (https://weblogo.berkeley.edu/).

## Supplementary Material

Appendix 01 (PDF)

## Data Availability

Crystallographic data and coordinates data have been deposited in rcsb.org (9DIE (73), 9OZO (74), and 9OZS (75)).
