# Supplementary material for: Spider venom phospholipase D toxin structure: Interfacial binding site, mechanism, activation, and head group preference
Source: Proc Natl Acad Sci U S A. 2026 Apr 6;123(15):e2513997123. doi: 10.1073/pnas.2513997123 (PMC13079978; doi:10.1073/pnas.2513997123)
Supplement: Supplementary file 1 — Appendix 01 (PDF) [file pnas.2513997123.sapp.pdf]

Table S1

Crystallographic data for St_βIB1i complexed with d17:1/12:0 CPE substrate and/or CCP product, from crystals grown over different incubation periods

	6 day	2 day	3 week
Data Measurement			
X-ray source	Gallium	Gallium	Gallium
Wavelength (Å)	1.34	1.34	1.34
Resolution (Å)	24.4 – 1.85	24.4 – 2.20	24.4 – 2.60
Observed reflections	503931	219316	254427
Unique reflections	76317	45116	28219
Multiplicity	6.6	4.9	9.0
Completeness (%)[a]	99.9 (100)	99.8 (99.4)	99.8 (100)
$I/\sigma(I)$[a]	9.6 (1.6)	7.1 (1.5)	4.1 (1.9)
R_{merge}[a]	0.17 (1.8)	0.16 (0.66)	0.40 (1.2)
Structure Refinement			
R_{cryst}/R_{free}[b]	0.12 / 0.17	0.15 / 0.20	0.15 / 0.21
rmsd bonds (Å)	0.009	0.007	0.008
rmsd angles (°)	1.77	1.60	1.76
Ramachandran outliers	0	0	0
PDB entry	9DIE	9OZO	9OZS

[a]Overall (outer shell). [b]Calculated from 5% of the data excluded from refinement.

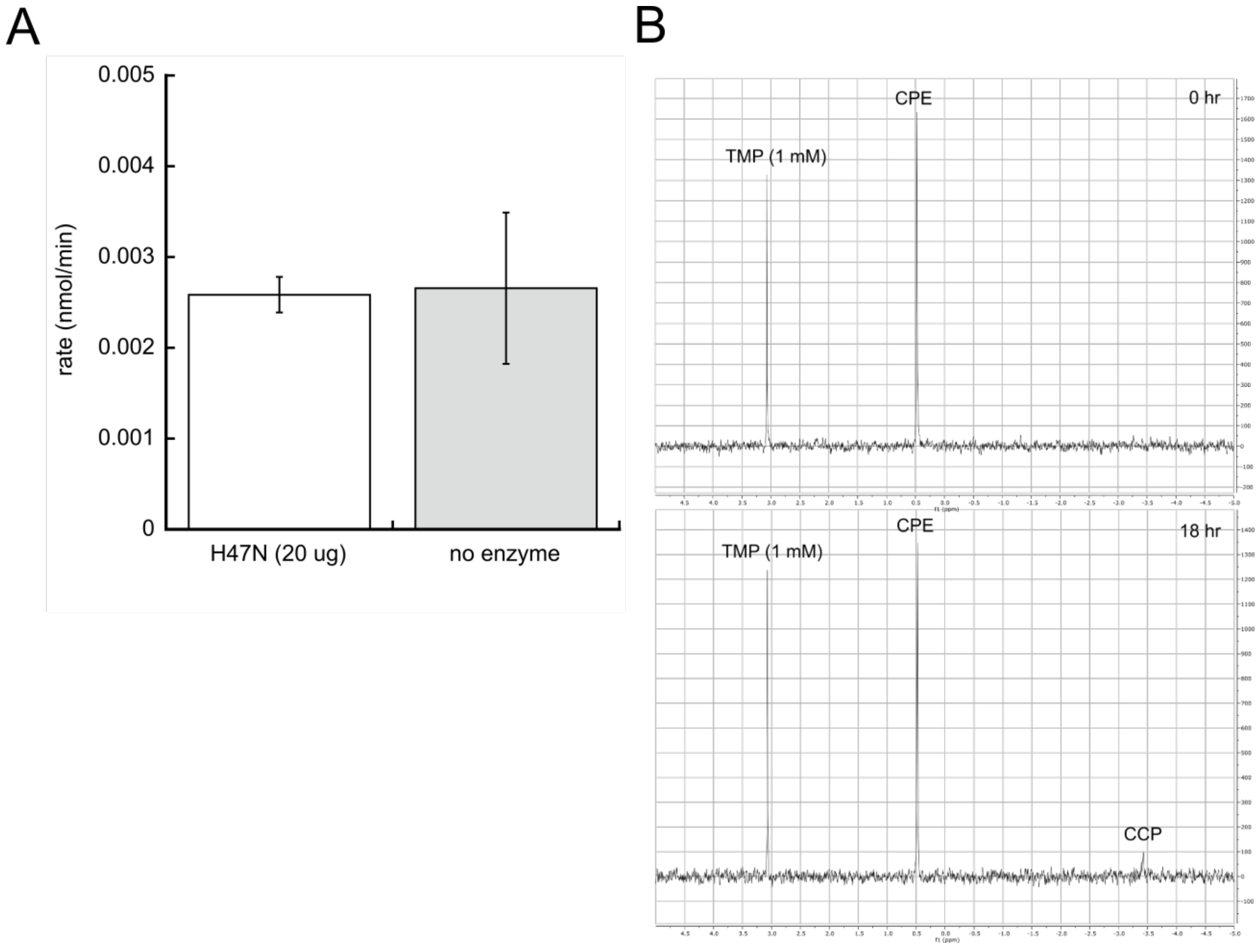

Figure S1. Residual enzymatic activity of an St_β1B1i H47N variant. (*A*) Head group release assay at 200 μM initial CPE concentration in 0.1% Triton X-100, 0.1 M Tris (pH 7.4), 10 mM $MgCl_2$, 37 °C. Data represent mean ± SEM (n=3). In this assay, H47N (at 20 μg) is indistinguishable from a no-enzyme control (P=0.92*,* determined by Student's t test), while wild type (per 1 μg) is about 70x faster (see Figure 3B). (*B*) ^{31}P NMR assay using 20 μg H47N enzyme at 2.2 mM initial CPE concentration solubilized in 50 mg/mL CHAPS, 0.1 M Tris (pH 7.4), 10 mM $MgCl_2$, 37 °C. After 18 h, 0.2 mM CCP product has formed as judged by peak fitting in MestreNova, referenced to a trimethyl phosphate (TMP) standard at 1 mM concentration. This concentration of product corresponds to an approximate initial rate of 0.01 nmol/min•μg, compared to >200 nmol/min•μg measured for the wild-type protein under comparable conditions (see Figure 5D). Significant residual activity for H47N appears only in the NMR assay, perhaps due to the higher initial substrate concentration and the extremely fast rate of the wild-type protein in CHAPS micelles (see Figure 5D).

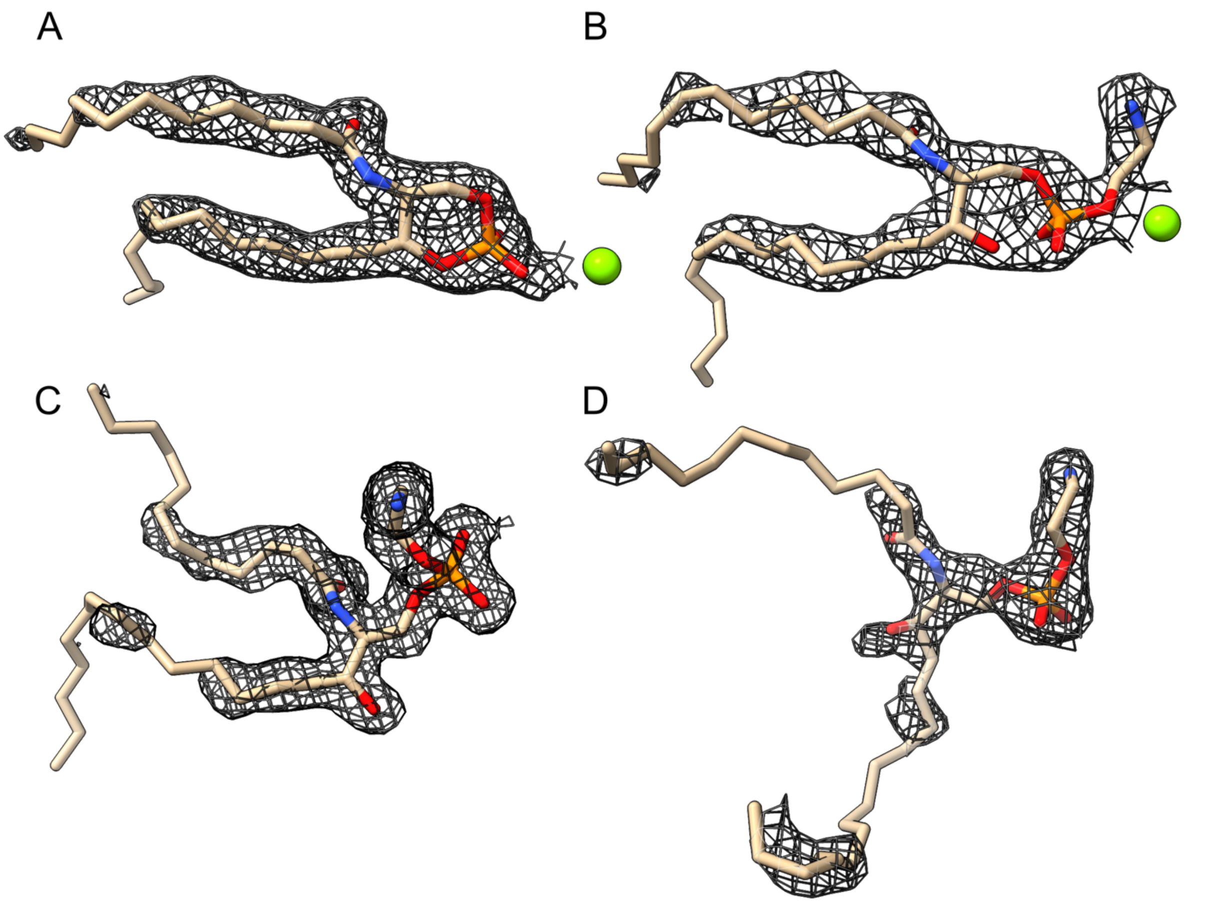

Figure S2. Lipid electron density. 2Fo-Fc density for *A*) CCP bound in the active site of chain B of the 1.85 Å (6 day growth) structure. *B*) CPE modeled in the active site of chain B of the 2.6 Å (3 week growth) structure. *C*) CPE in the triloop site of chain B of the 1.85 Å (6 day growth) structure, *D*) CPE in the β2α2/α1 site of chain A of the 2.2 Å (2 day growth) structure. Surface is contoured at 1 σ. *A* and *B* also show active site magnesium (green).

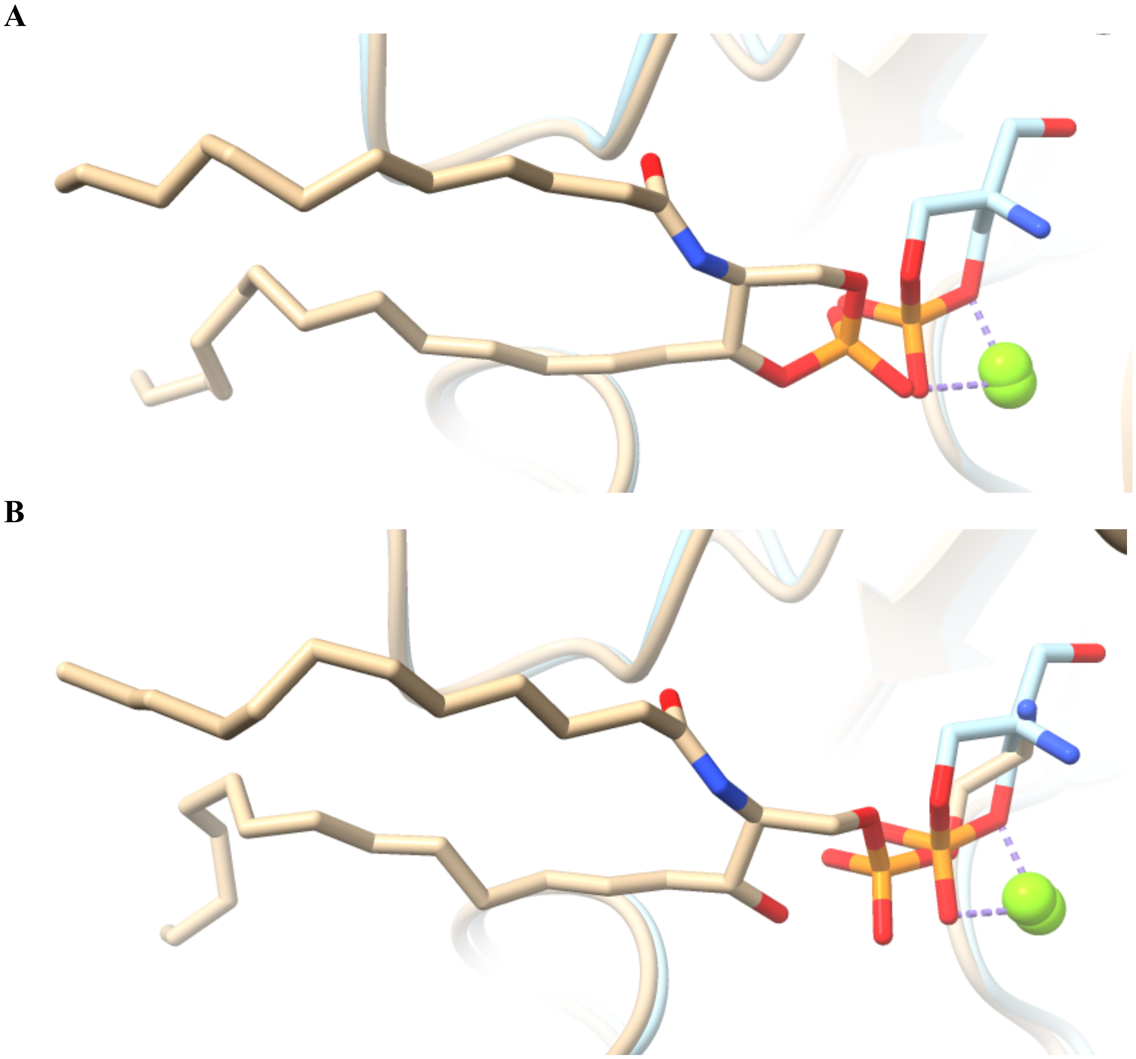

Figure S3. Comparison of CCP/CPE binding mode in St_βIB1i H47N to cyclic phosphate modeled in the reprocessed Li_αIA1 structure. The orientation of the adventitious cyclic phosphate moiety in Li_αIA1 (light blue), putatively derived from a combination of Tris and phosphate, is distinct and effectively reversed in orientation relative to *A*) the CCP lipid product bound to St_βIB1i (tan). In fact, it more closely reflects *B*) our observed substrate-binding mode, with a portion of the cyclic phosphate moiety roughly mimicking the ethanolamine leaving group of CPE substrate bound to St_βIB1i (light green).

A

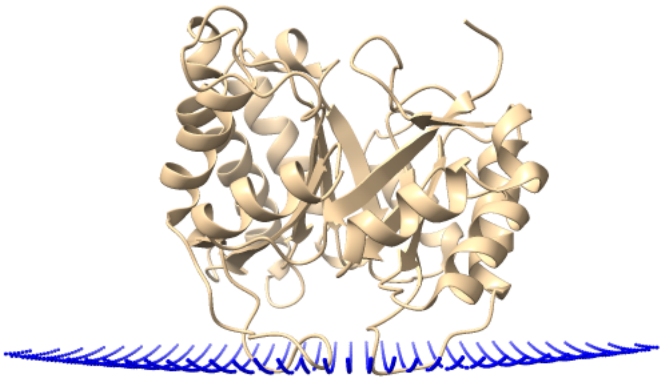

B

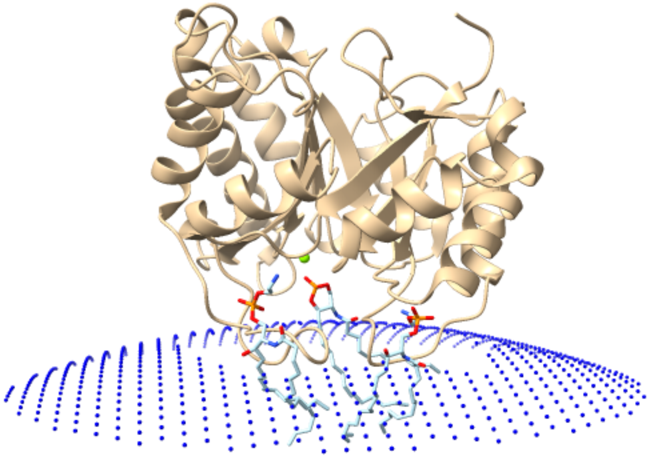

Figure S4. Predictions of St_ β1B1i membrane association by PPM 3.0. (A) St_ β1B1 H47N 1.85 Å structure (9DIE, chain A) bound to undefined membrane, with no bound lipids included in the interfacial prediction; (B) with bound lipids included. As with previous molecular dynamics calculations (compare to Figure 4A), the predicted interfaces center around the β2α2 and β6α6 loops, albeit with less of a lean toward the α7 and α8 helices. Inclusion of bound lipids from the structure imparts a predicted negative curvature (80 Å intrinsic radius of curvature) to the membrane.

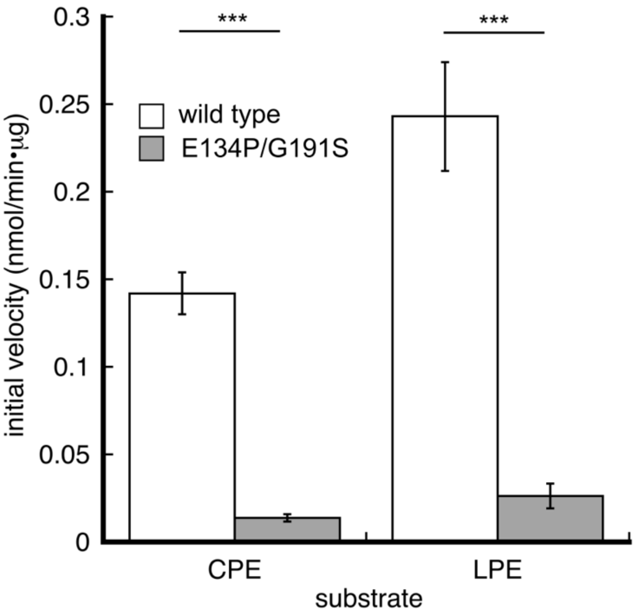

Figure S5. Effect of an E134P/G191S mutation on activity against lipids with PEtn head groups. Amplex enzyme-coupled head-group release assay with nominal 200 μM porcine brain CPE or synthetic 14:0 LPE as substrate, in 0.1 M Tris (pH 7.4), 10 mM $MgCl_2$, 0.1% Triton X-100, 37 °C. Data represent mean ± S.E.M (n=5). ****P* < 0.0005 as determined by Student's t test.

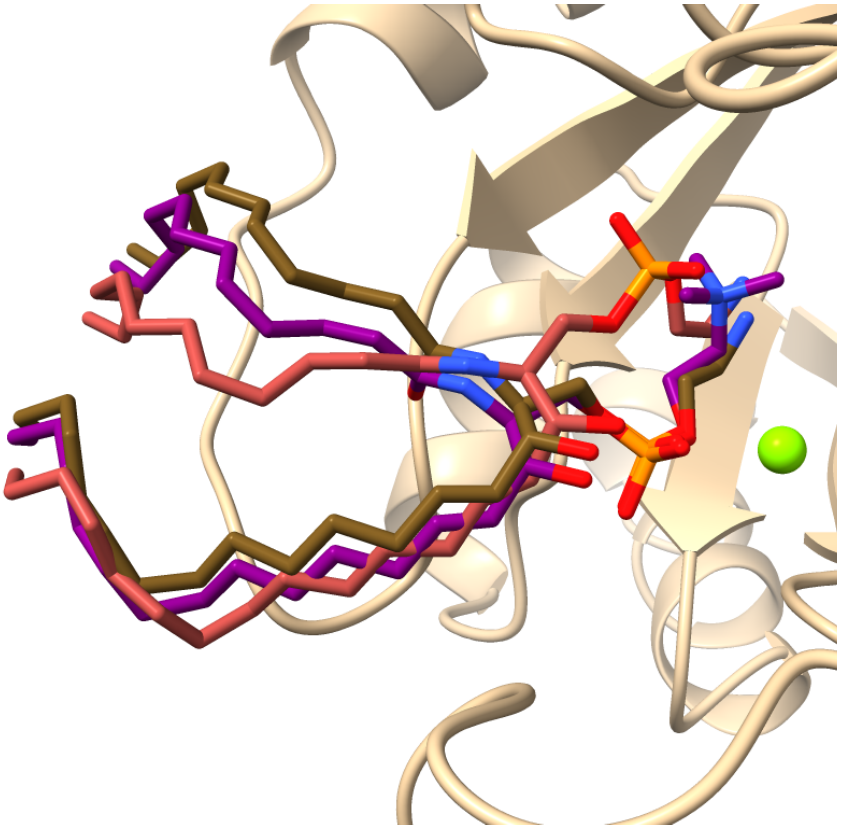

Figure S6. Representative low-energy poses from docking studies. Lowest energy pose of SM (-8.7 kcal/mol vs. -8.2 for next lowest) docked to Li_α1A1 (purple); the comparable pose for CPE docked to St_β1B1i (brown); and a second pose type commonly recovered as lowest or second lowest energy in docking of CPE to St_ β1B1i (brick red), in which the phosphate is pedaled outward away from the magnesium. The second pose type was approximately equal to or slightly lower in energy than the first (-8.5 kcal/mol vs. -8.3 kcal/mol on average; 0.0-0.5 kcal/mol lower for individual runs), depending on which chain was used as the receptor, whether or not sodium was present in the active site, and which alternate conformers of Asp-91 and Met-246 were used. Given the comparable energy, the first CPE pose was considered more reasonable based on its similarity to the lowest energy SM pose with Li_α1A1, as well as its closer similarity to the experimental binding mode of CPE observed with St_ β1B1i H47N (see Figure 6A).

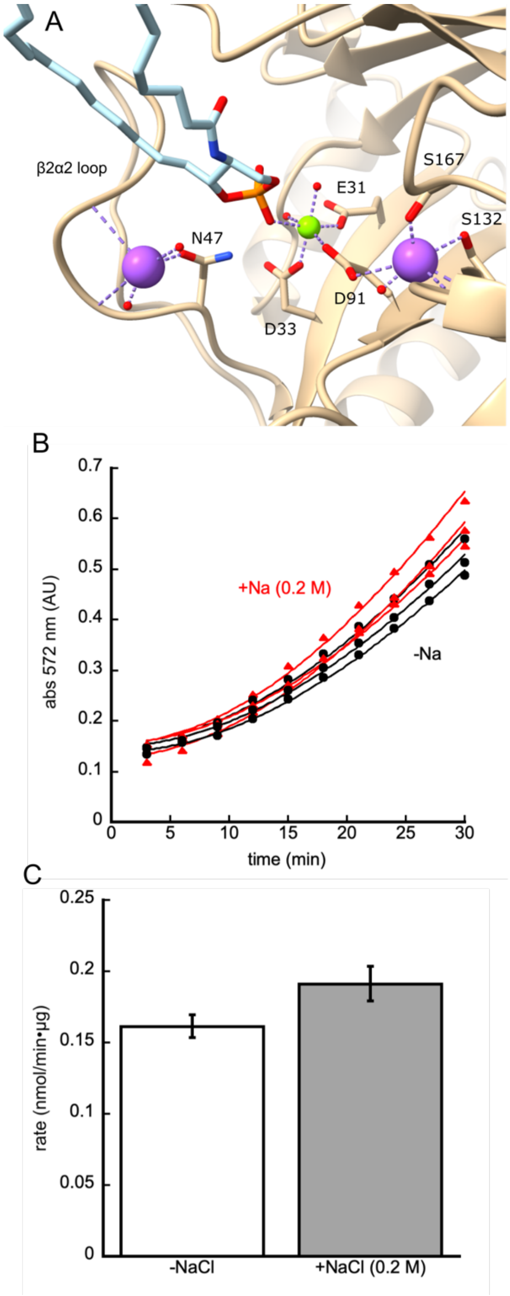

Figure S7. Sodium ions in or near the St_βIB1i H47N active site. (*A*) Two sodium ions (purple) modeled in the 1.85Å product-bound structure, one bound by the β2α2 loop, and another deep in the active-site pocket coordinated by the side chains of D91, S132, S167 along with backbone carbonyl O atoms from S132 and L133. (*B*) Head-group release assays in triplicate using 200 μM CPE substrate, with (red) or without (black) 200 mM sodium chloride. Release of ethanolamine is indirectly detected by secondary formation of resorufin, which absorbs at 572 nm. The nonlinear appearance of the curve is due to the secondary enzymes being partially kinetically limiting. The initial rate for the primary enzymatic reaction is extracted by fitting the reaction as two sequential reactions (see Methods). (*C*) Mean initial rates from the experiments shown in *B*, ± S.E.M. (n=3). *P* = 0.10 as determined by Student t-test, indicating that a significant effect is not demonstrated.
